# Pharmacological Evaluation of Naproxen Metal Complexes on Antinociceptive, Anxiolytic, CNS Depressant, and Hypoglycemic Properties

**DOI:** 10.1155/2016/3040724

**Published:** 2016-07-11

**Authors:** Md. Sharif Hasan, Narhari Das, Zobaer Al Mahmud, S. M. Abdur Rahman

**Affiliations:** Department of Clinical Pharmacy & Pharmacology, Faculty of Pharmacy, University of Dhaka, Dhaka 1000, Bangladesh

## Abstract

*Purpose*. The present study was designed to investigate the antinociceptive, anxiolytic, CNS depressant, and hypoglycemic effects of the naproxen metal complexes.* Methods*. The antinociceptive activity was evaluated by acetic acid-induced writhing method and radiant heat tail-flick method while anxiolytic activity was evaluated by elevated plus maze model. The CNS depressant activity of naproxen metal complexes was assessed using phenobarbitone-induced sleeping time test and the hypoglycemic test was performed using oral glucose tolerance test.* Results*. Metal complexes significantly (*P* < 0.001) reduced the number of abdominal muscle contractions induced by 0.7% acetic acid solution in a dose dependent manner. At the dose of 25 mg/kg body weight p.o. copper, cobalt, and zinc complexes exhibited higher antinociceptive activity having 59.15%, 60.56%, and 57.75% of writhing inhibition, respectively, than the parent ligand naproxen (54.93%). In tail-flick test, at both doses of 25 and 50 mg/kg, the copper, cobalt, silver, and zinc complexes showed higher antinociceptive activity after 90 minutes than the parent drug naproxen. In elevated plus maze (EPM) model the cobalt and zinc complexes of naproxen showed significant anxiolytic effects in dose dependent manner, while the copper, cobalt, and zinc complexes showed significant CNS depressant and hypoglycemic activity.* Conclusion*. The present study demonstrated that copper, cobalt, and zinc complexes possess higher antinociceptive, anxiolytic, CNS depressant, and hypoglycemic properties than the parent ligand.

## 1. Introduction

Recent findings on the chemical and biochemical activity of metal complexes play an essential role in agriculture and pharmaceutical and industrial chemistry [1]. In therapeutics, the use of metal complexes with traditional drugs as therapeutic agents for treatment of different diseases has been extensively studied [2–6]. As they generally possess different mechanisms of activity from the organic compounds, the development of metal complexes provides an alternative route of novel drug delivery system [7]. Very recent works on metal complexes have proved that binding of a drug to metalloelement enhances its activity and in many cases the complex possesses even such activity that the parent compound does not have any of those [8]. Thus we have been motivated to study metal binding properties of naproxen derivatives with different transition metal ions and analyzed its different biological properties for the sake of getting any new possibilities of using naproxen metal complexes for different therapeutic purposes.

Naproxen, an NSAID, is extensively used in the clinical treatment of arthritis and different pain states. According to previous studies, metal ions play a significant role in different biological processes and some diseases progression happens due to the lack of many transition metallic elements vital to life [9].

To the best of our knowledge and available literature on the subject no work has been done on all of these properties of naproxen metal complexes. In the present study we report naproxen metal complexes with the* in vivo* antinociceptive, anxiolytic, CNS depressant, and hypoglycemic activities.

## 2. Materials and Methods

### 2.1. General Procedure for Synthesis of Transition Metal Complexes of Naproxen

A detailed procedure of synthesis of naproxen metal complexes was described in our previous paper [10].

### 2.2. Chemicals and Reagents

Morphine and diazepam were collected from Popular Pharmaceuticals and Gonoshasthaya Pharmaceuticals Ltd., Dhaka, Bangladesh. Acetic acid was obtained from Merck, Germany. All other chemicals were obtained commercially and were of analytical grade.

## 3. Biological Evaluation

### 3.1. Experimental Animals

Swiss-albino mice (20–25 gm) of either sex, aged 4-5 weeks, obtained from the Animal Resource Branch of Jahangirnagar University, Dhaka, Bangladesh, were used for the experiment. They were housed in the standard polypropylene cages (30 × 20 × 13 cm) and kept under standard laboratory conditions (relative humidity 55–60%, room temperature 23 ± 2°C, and 12-hour light/dark cycle) for 7 days prior to performing the experiments. They were fed standard formulated rodent food and water* ad libitum*. All experimental protocols were approved by Faculty of Pharmacy Ethics Committee, University of Dhaka.

### 3.2. Antinociceptive Activity Study

#### 3.2.1. Peripheral Antinociceptive Activity Study

Antinociceptive activity was evaluated by the acetic acid-induced writhing method in mice [11]. The writhes were induced by intraperitoneal injection of 0.7% acetic acid (v/v) (10 mL/kg). Two different doses (25 and 50 mg/kg) of the naproxen metal derivatives were administered orally to different groups of 5 animals each, 30 min before chemical stimulus. The number of writhes during the following 15 min period was observed after acetic acid injection. Antinociception was expressed as the reduction of the number of abdominal constrictions between control animals (acetic acid treated mice) and mice pretreated with the naproxen metal derivatives. Naproxen (25 mg/kg) was used as standard.

#### 3.2.2. Central Antinociceptive Activity Study

Antinociceptive activity was evaluated by radiant heat tail-flick method [12]. Test samples and control were given orally by means of a feeding needle to the mice at zero hours. A 30-minute interval was given to ensure proper absorption of the administered substances. After 30 minutes, the tail-flick time was measured by analgesiometer (Medicraft, India). Morphine (2 mg/kg, s.c.) was used as standard. The strength of the current passing through the naked nichrome wire was kept constant at 3 Amps. The tail skin was kept at a distance of 1.5 cm from the heat source. The radiant heat (55 ± 2°C) in the tail was applied and maintained at 2.5 cm measured from the root of the tail. In order to avoid the tissue damage, the cut of reaction time was kept at 16 sec.

### 3.3. Anxiolytic Activity Study

#### 3.3.1. Elevated Plus Maze (EPM) Model

The plus maze apparatus consisted of two open arms, measuring 16 × 5 cm, and two closed arms, measuring 16 × 5 × 12 cm, connected to a central platform (5 × 5 cm). The maze was elevated to a height of 25 cm above the floor. Two different doses (25 and 50 mg/kg) of the naproxen metal derivatives were administered orally to different groups of 6 animals each, 45 min prior to experiment. Each mouse was placed individually at the center of elevated plus maze with its head facing toward an open arm and observed for 5 min to record the number of entries into open arm and closed arm and time spent in each arm [13]. Diazepam (2 mg/kg, p.o.) was used as standard.

#### 3.3.2. Central Nervous System (CNS) Depressant Activity Study

Naproxen metal chelates were assessed for effect on the CNS using phenobarbitone-induced sleeping time test in mice [14]. Thirty min after the oral administration of naproxen and its derived complexes (25 mg/kg), vehicle control (1% Tween-80 solution in saline), and intraperitoneal injection of diazepam (1 mg/kg), all mice were injected with phenobarbitone (25 mg/kg, i.p.). The animals were observed for the latent period (time between phenobarbitone administration and loss of righting reflex) and duration of sleep (time between the loss and recovery of righting reflex).

### 3.4. Hypoglycemic Activity Study

The test was performed using a slight modification [15]. The animals were weighed and randomly divided into eight groups consisting of 6 mice in each group. At zero hours fasting blood glucose level from each group was measured from tail vein just prior to glucose administration by using glucometer (Braun G-423 S from Hong Kong) and glucose oxidase-peroxidase reactive strips. To measure the blood glucose level, tail tip of mice was cut with a sharp blade and then little amount of blood was collected and exposed to the touch of glucose test strips. Within seconds blood glucose level was visualized. Nebanol (Bacitracin) ointment was applied on the wound to avoid infection. Then the samples were administered (0.1% saline as control, metformin as standard and naproxen metal chelates) using oral feeding needle. After 1 hour, 2 hours, and 3 hours, blood was collected in the same procedure and blood glucose level was measured to see the hypoglycemic effect of the test sample compared to control and standard groups.

### 3.5. Statistical Analysis

All the values are expressed as mean ± standard error of the mean (SEM). One-way ANOVA followed by Dunnett's *t*-test was used to determine a significant difference between control groups and experimental groups. *P* ≤ 0.001 was considered to be statistically significant.

## 4. Results

### 4.1. Characterization of Metal Complexes of Naproxen

Physical, analytical, and thermal properties, NMR spectra, FTIR spectra, scanning electron microscopy, and HPLC study of naproxen metal complexes were described by Hasan et al. [10].

### 4.2. Peripheral Antinociceptive Activity Study

Naproxen and its metal chelates were subjected to screening for antinociceptive activity by acetic acid-induced writhing inhibition method. Metal complexes effectively reduced the number of abdominal muscle contractions induced by 0.7% acetic acid solution in a dose dependent manner as shown in Table 1. At the dose of 25 mg/kg body weight, copper, cobalt, and zinc complexes showed significant (*P* < 0.001) antinociceptive activity having 59.15%, 60.56%, and 57.75% of inhibition, respectively, compared to the standard naproxen (54.93%). On the other hand, copper, cobalt, and zinc complexes significantly (*P* < 0.001) demonstrated 73.24%, 74.65%, and 67.60% inhibition of writhing at 50 mg/kg body weight.

### 4.3. Central Antinociceptive Activity Study

In the tail-flick model, reaction time was increased significantly for the test samples and standard drug when compared to the predrug reaction time (control group) 30 minutes after drug administration and thus it appears that the test sample inhibits predominantly the peripheral pain mechanism. Naproxen metal chelates effectively elongated the reaction time in a dose dependent manner. At low doses (25 mg/kg), copper, silver, and zinc complexes showed significant antinociceptive activity after 90 minutes having 102.61%, 101.31%, and 99.02% elongation of reaction time, respectively, compared to the standard morphine (113.40%). The result was found statistically significant as shown in Table 2.

### 4.4. Elevated Plus Maze (EPM) Model

Naproxen and its cobalt and zinc complexes significantly increase time spent in the open arm having 11.67 seconds, 13.5 seconds, and 12.67 seconds, respectively (*P* < 0.001). However, they did not improve the number of entries in the open arm as shown in Table 3.

### 4.5. CNS Depressant Activity Study

In phenobarbitone-induced sleeping time test, the naproxen at a dose of 25 mg/kg significantly (*P* < 0.001) prolonged the duration of sleeping time (143.33 min) in test animals as compared to control (120.5 min) which was comparable to that of standard drug diazepam (182.0 min; *P* < 0.001). Its metal chelates also increased duration of sleep compared to control. However, they did not show any increase in duration of sleep compared to naproxen except naproxen-copper complex. The overall result of CNS depressant property of naproxen and its metal chelates is shown in Table 4.

### 4.6. Hypoglycemic Activity Study

The results found from naproxen and its derived metal complexes showed significant blood glucose lowering activity as shown in Table 5. The test was performed by taking the samples at the doses 25 mg/kg body weight. Copper, cobalt, and zinc complexes of naproxen showed significant decrease of plasma glucose level. After 3 hours, the plasma glucose levels of copper, cobalt, and zinc complexes of naproxen were 4.72 mmol/L, 4.60 mmol/L, and 4.17 mmol/L, respectively (*P* < 0.001).

## 5. Discussion

In this study, antinociceptive activity was evaluated by two distinctly separate methods. For studying the peripheral (nonnarcotic) antinociceptive mechanism of naproxen metal complexes, acetic acid-induced writhing test in mice is the most suitable and widely used method. Here, peritoneal administration of acetic acid (0.7%) causes localised inflammation in mice. Following inflammation, there is biogenesis of prostaglandins from cyclooxygenase pathway and leukotrienes from lipoxygenase pathway. The released prostaglandins, mainly prostacyclin (PGI2) and, to a lesser extent, prostaglandin E, have been held responsible for pain sensation and abnormal contraction of mice body which is termed as “writhing” [16]. In this experiment, naproxen and its different complexes significantly (*P* < 0.001) lowered the number of writhes which was comparable with that obtained by the standard naproxen. Therefore, the results of the acetic acid-induced writhing strongly suggest that the mechanism of naproxen and its complexes may be linked partly to the inhibition of lipoxygenase and/or cyclooxygenase in the peripheral tissue, thereby reducing PGE2 synthesis and interfering with the mechanism of transduction in the primary afferent nociceptor. Furthermore, we complemented the analgesic effect by using another nociceptive technique that made our findings more convincing [17, 18]. To investigate whether naproxen and its different complexes have true analgesic potential, the radiant heat tail-flick method was also used. This method measures the complex response to a noninflammatory, acute nociceptive input and is one of the models normally used for studying central (narcotic) antinociceptive activity. As the reaction time was increased significantly for the test samples and standard drug in comparison with the predrug reaction time (control group) at 90 min after drug administration, the findings ensure the predominant inhibition of the central pain mechanism by the test samples.

Both in the acetic acid-induced writhing and in tail-flick methods, naproxen and its complexes (25 and 50 mg/kg) and standard drugs showed significant results as compared with the control group. As the naproxen metal complexes appeared to be active in both animal models of nociception, they may possess peripherally and centrally acting compounds for its antinociceptive action.

The EPM is an animal model of anxiety useful to predict the anxiolytic effects of benzodiazepines (BZD) [19]. Cyclooxygenase (COX) is reported to play an important role in pathogenesis of various neurodegenerative disorders including stroke and seizures [20]. Therefore, the use of COX inhibitors may be a useful neuroprotective strategy in the treatment of stress. Very recently, a new class of cyclooxygenase inhibitors with antianxiety activity has been reported [21]. Inhibitors that block cyclooxygenase-2-dependent endocannabinoid oxygenation increase endocannabinoid tone and reduce anxiety in mice. Therefore, it may be concluded that naproxen and cobalt and zinc complexes of naproxen have such inhibitory action, thus showing antianxiety activity.

Previous research works of different times were also very supportive of our findings. Naproxen has the ability to attenuate the immobilization stress-induced behavioural and biochemical alterations [20]. In case of naproxen-zinc complex, zinc has been found to be associated with GABA and glutamate regulation, particularly through anxiolytic activity, modulating GABAergic inhibition and seizure susceptibility [22, 23]. We also found naproxen-cobalt complex as anxiolytic agent; however other complexes do not have any antianxiety effects. In case of copper, it was observed that high copper concentration in plasma was found in individuals with anxiety [23]. So it is expected that there is antianxiety activity of naproxen-copper complex.

Naproxen metal chelates effectively elongated the total sleeping time as well as duration of sleeping time. Copper, cobalt, iron, and zinc complexes showed significant depressant activity. All of these complexes were found to have anticonvulsant activities [24]; thus they might be able to provide depressant activity in this regard. Other studies [25] showed that cobalt complexes had higher central nervous system (CNS) depressant activity and also more toxicity as compared to the ligand.

NSAIDs are known to penetrate the central nervous system [26] and have been shown to have direct effects on various neuronal (and nonneuronal) ion channels including nonselective cation channels [27, 28] and chloride channels [29, 30]. More recently, a number of NSAIDs have also been shown to modulate rat brain *γ*-aminobutyric acid type-A (GABA_A_) receptors expressed in* Xenopus* oocytes [31]. Moreover, a preliminary study by Halliwel et al. [32] demonstrated a marked positive modulation of GABA-mediated currents recorded in rat hippocampal neurons by mefenamic acid especially.

GABA may also play a role in many other physiological and behavioural processes such as arousal, sexual behaviour, coma, stress, anxiety, depression, memory, thermal regulation, muscle relaxation, and sleep. Many anaesthetic agents have been shown to produce sedation and anaesthesia probably by enhancing GABA-mediated synaptic transmission [33].

It is well established that antagonists of the GABA_A_ receptor, such as bicuculline and picrotoxin, are proconvulsant drugs [34], and positive allosteric modulators of the GABA_A_ receptor such as alphaxalone, propofol, and diazepam enhance the inhibitory actions of *γ*-aminobutyric acid (GABA) and thereby reduce neuronal excitation. It is possible, therefore, that some of the actions of NSAIDs may be mediated through direct effects on the CNS. In particular, coma and/or convulsions suggest an interaction between these drugs and neuronal ligand gated ion channels. It has been reported that *α*1 GABA_A_ receptors mediate the sedative and anticonvulsant actions of diazepine [35]. Based on this document it can be stated that naproxen metal chelates might bind nonselectively with the *α*1 GABA_A_ receptors, thus providing depressant activity.

Metal complexes of various organic ligands with copper [36, 37], cobalt [38], and zinc [39] have been reported to exhibit insulin-mimetic or enhancing properties both* in vitro* and* in vivo*. The insulin-mimetic activity of these complexes was evaluated by means of* in vitro* measurements of the inhibition of free fatty acid (FFA) release from epinephrine-treated, isolated rat adipocytes [36].

Elevated plasma levels of free fatty acids (FFAs) are known to cause peripheral (muscle) insulin resistance [40] by inhibiting insulin-stimulated glucose uptake and glycogen synthesis. FFAs also cause hepatic insulin resistance by inhibiting insulin-mediated suppression of glycogenolysis. FFAs have recently been shown to activate the IkappaB/NFkappaB pathway which is involved in many inflammatory processes. Thus inhibiting FFA by naproxen metal chelates may also find another pathway for showing anti-inflammatory activity.

This experiment of naproxen metal chelates showed better hypoglycemic activity than control group assuming that the respective metals provide the hypoglycemic effects of their respective mechanism of actions. However, the silver and iron metal chelates had no hypoglycemic activity.

## 6. Conclusion

Search for drugs of higher efficacy and lower toxicity is a never-ending effort. For the first time, we have reported the* in vivo* activity of naproxen metal complexes. From these investigations, it may be concluded that naproxen metal chelates showed significant antinociceptive, CNS depressant, and hypoglycemic properties while naproxen and its cobalt and zinc chelates have anxiolytic effects. Among all complexes, the copper, cobalt, and zinc complexes possess higher antinociceptive, anxiolytic, CNS depressant, and hypoglycemic properties than the parent ligand, naproxen. However, studies are required on higher animal model and subsequently on human subjects to prove clinical efficacy of naproxen as an antinociceptive and anxiolytic and further research is essential to find out the possible side effects that it may provide because of central metal of chelation and its principles responsible for such activity.

## Figures and Tables

**Table 1:** Effects of naproxen and its metal chelates on acetic acid-induced writhing response in mice.

Group	Dose	Number of writhes (mean ± SEM)	% inhibition of writhing
Control	10 mL/kg	14.2 ± 1.07	—
Standard	25 mg/kg	6.4 ± 0.68^*∗*^	54.93
Naproxen-copper complex	25 mg/kg	5.8 ± 0.86^*∗*^	59.15
Naproxen-copper complex	50 mg/kg	3.8 ± 0.58^*∗*^	73.24
Naproxen-cobalt complex	25 mg/kg	5.6 ± 0.68^*∗*^	60.56
Naproxen-cobalt complex	50 mg/kg	3.6 ± 0.51^*∗*^	74.65
Naproxen-iron complex	25 mg/kg	7.4 ± 0.40^*∗*^	47.89
Naproxen-iron complex	50 mg/kg	5.6 ± 0.51^*∗*^	60.56
Naproxen-silver complex	25 mg/kg	6.6 ± 0.93^*∗*^	53.52
Naproxen-silver complex	50 mg/kg	4.2 ± 0.73^*∗*^	70.42
Naproxen-zinc complex	25 mg/kg	6.0 ± 0.31^*∗*^	57.75
Naproxen-zinc complex	50 mg/kg	4.6 ± 0.68^*∗*^	67.60

Each value represents the mean ± SEM, *n* = 5. ^*∗*^ *P* < 0.001 was considered significant compared with control.

**Table 2:** Central antinociceptive activity of naproxen and its metal chelates at different time intervals.

Group	Dose (mg/kg)	Reaction time (sec)		
		30 min (% elongation)	60 min (% elongation)	90 min (% elongation)
Control	—	6.38 ± 0.34	5.86 ± 0.46	6.12 ± 0.23
Morphine	02	18.6 ± 0.59^*∗*^ (191.54)	15.86 ± 0.28^*∗*^ (170.65)	13.06 ± 0.72^*∗*^ (113.40)
Naproxen	25	9.97 ± 0.52^*∗*^ (56.27)	11.06 ± 0.45^*∗*^ (88.74)	10.46 ± 0.70^*∗*^ (70.92)
Naproxen	50	12.12 ± 0.62^*∗*^ (89.97)	11.94 ± 0.80^*∗*^ (103.75)	13.3 ± 0.42^*∗*^ (117.32)
Naproxen-copper complex	25	10.8 ± 0.33^*∗*^ (69.28)	10.8 ± 0.44^*∗*^ (84.30)	12.4 ± 0.34^*∗*^ (102.61)
Naproxen-copper complex	50	14.62 ± 0.42^*∗*^ (129.15)	14.8 ± 0.35^*∗*^ (152.56)	14.52 ± 0.49^*∗*^ (137.25)
Naproxen-cobalt complex	25	11.02 ± 0.48^*∗*^ (72.73)	11.32 ± 0.53^*∗*^ (93.17)	11.96 ± 0.50^*∗*^ (95.42)
Naproxen-cobalt complex	50	13.38 ± 0.34^*∗*^ (109.72)	15.3 ± 0.86^*∗*^ (161.09)	14.86 ± 0.44^*∗*^ (142.81)
Naproxen-iron complex	25	9.84 ± 0.79^*∗*^ (54.23)	10.78 ± 0.61^*∗*^ (83.96)	11.04 ± 0.52^*∗*^ (80.39)
Naproxen-iron complex	50	13.5 ± 0.84^*∗*^ (111.60)	13.64 ± 0.69^*∗*^ (132.76)	12.74 ± 0.87^*∗*^ (108.17)
Naproxen-silver complex	25	10.84 ± 0.55^*∗*^ (69.91)	11.06 ± 0.61^*∗*^ (88.74)	12.32 ± 0.45^*∗*^ (101.31)
Naproxen-silver complex	50	13.8 ± 0.58^*∗*^ (116.30)	14.18 ± 0.45^*∗*^ (141.98)	13.6 ± 0.42^*∗*^ (122.22)
Naproxen-zinc complex	25	12.48 ± 0.29^*∗*^ (95.61)	12.86 ± 0.51^*∗*^ (119.45)	12.18 ± 0.54^*∗*^ (99.02)
Naproxen-zinc complex	50	15.38 ± 0.45^*∗*^ (141.07)	15.44 ± 0.47^*∗*^ (163.48)	15.12 ± 0.31^*∗*^ (147.06)

Each value represents the mean ± SEM (*n* = 5). ^*∗*^ *P* < 0.001 was considered significant compared with control.

**Table 3:** Effect of naproxen and its metal complexes on the number of entries and time spent in elevated plus maze in acute study.

Group	Dose (mg/kg)	Observed parameters				
		Open arm entries	Closed arm entries	Total entries	Time spent in open arms	Time spent in closed arms
Control	—	2.3 ± 0.21	2.83 ± 0.40	5.17 ± 0.47	6.83 ± 0.47	293.17 ± 0.47
Diazepam	2	5.17 ± 0.54^*∗∗*^	4.5 ± 0.56^*∗∗*^	9.67 ± 1.02^*∗∗*^	17.83 ± 0.94^**∗∗**^	282.17 ± 0.94^**∗∗**^
Naproxen	25	3.17 ± 0.30	4.0 ± 0.36	7.17 ± 0.54	11.67 ± 0.80^*∗*^	288.33 ± 0.80^*∗*^
N-Cu	25	2.83 ± 0.47	3.33 ± 0.42	7.17 ± 0.47	7.67 ± 0.80	292.33 ± 0.80
N-Co	25	3.33 ± 0.33	3.67 ± 0.33	7.0 ± 0.57	13.5 ± 0.99^*∗∗*^	286.5 ± 1.07^**∗∗**^
N-Fe	25	1.83 ± 0.30	4.0 ± 0.36	5.83 ± 0.16	4.0 ± 0.63	296.0 ± 0.63
N-Ag	25	1.5 ± 0.34	3.83 ± 0.33	5.17 ± 0.40	3.67 ± 0.71	296.33 ± 0.71
N-Zn	25	3.17 ± 0.49	3.5 ± 0.42	6.67 ± 0.60	12.67 ± 1.33^**∗∗**^	287.33 ± 1.33^**∗∗**^

Each value represents the mean ± SEM, *n* = 6. ^*∗*^ *P* < 0.01 and ^*∗∗*^ *P* < 0.001 were considered significant compared with control.

**Table 4:** Effect of naproxen metal complexes on phenobarbitone sodium-induced sleep.

Group	Dose (mg/kg)	Parameters observed		
		Time of onset of sleep (min)	Total sleeping time (min)	Duration of sleep (min)
Control	—	16.67 ± 0.71	137.17 ± 1.7	120.50 ± 1.8
Naproxen	25	13.83 ± 0.60	157.17 ± 3.6^*∗∗*^	143.33 ± 3.7^*∗∗*^
Naproxen-copper complex	25	15.00 ± 0.85	172.00 ± 3.1^*∗∗*^	157.00 ± 3.7^*∗∗*^
Naproxen-cobalt complex	25	14.00 ± 0.60	154.17 ± 2.7^*∗∗*^	140.17 ± 3.2^*∗∗*^
Naproxen-iron complex	25	14.00 ± 0.96	153.83 ± 2.1^*∗∗*^	139.83 ± 2.1^*∗∗*^
Naproxen-silver complex	25	15.17 ± 1.2	138.00 ± 2.6	122.83 ± 3.6
Naproxen-zinc complex	25	13.33 ± 0.49^*∗*^	153.50 ± 2.5^*∗∗*^	140.17 ± 2.1^*∗∗*^
Diazepam	1	4.80 ± 0.37^*∗∗*^	186.80 ± 2.5^*∗∗*^	182.00 ± 3.11^*∗∗*^

Each value represents the mean ± SEM (*n* = 6). ^*∗*^ *P* < 0.05 and ^*∗∗*^ *P* < 0.001 compared with control.

**Table 5:** Effect of naproxen metal complexes on hypoglycemic property in mice.

Group	Dose (mg/kg)	Plasma glucose level in mmol/L			
		0 hours	1st hour	2nd hour	3rd hour
Control	—	5.98 ± 0.28	6 ± 0.15	5.9 ± 0.19	5.75 ± 0.17
Standard	50	6.05 ± 0.18	3.78 ± 0.13^*∗*^	3.6 ± 0.15^*∗*^	3.43 ± 0.11^*∗*^
Naproxen	25	5.72 ± 0.27	5.78 ± 0.15	5.58 ± 0.10	5.73 ± 0.19
Naproxen-copper complex	25	6.15 ± 0.19	4.37 ± 0.19^*∗*^	4.3 ± 0.05^*∗*^	4.72 ± 0.13^*∗*^
Naproxen-cobalt complex	25	5.73 ± 0.18	4.7 ± 0.17^*∗*^	4.5 ± 0.10^*∗*^	4.6 ± 0.14^*∗*^
Naproxen-iron complex	25	5.78 ± 0.22	5.58 ± 0.24	5.65 ± 0.20	5.7 ± 0.19
Naproxen-silver complex	25	5.8 ± 0.24	6.03 ± 0.13	5.82 ± 0.12	5.72 ± 0.18
Naproxen-zinc complex	25	5.22 ± 0.22	4.53 ± 0.18^*∗*^	4.17 ± 0.17^*∗*^	4.17 ± 0.14^*∗*^

Each value represents the mean ± SEM, *n* = 6.  ^*∗*^ *P* < 0.001 was considered significant compared with control.
